# A New Hybrid Possibilistic-Probabilistic Decision-Making Scheme for Classification

**DOI:** 10.3390/e23010067

**Published:** 2021-01-03

**Authors:** Basel Solaiman, Didier Guériot, Shaban Almouahed, Bassem Alsahwa, Éloi Bossé

**Affiliations:** Image & Information Processing Department (iTi), IMT-Atlantique, Technopôle Brest Iroise CS 83818, 29238 Brest, France; basel.solaiman@imt-atlantique.fr (B.S.); didier.gueriot@imt-atlantique.fr (D.G.); shaban.almouahed@bsb-marine.com (S.A.); bassems74@netcourrier.com (B.A.)

**Keywords:** possibility theory, possibilistic decision rule, possibilistic maximum likelihood, pattern classification, uncertainty, Bayesian decision, maximum *a posteriori*, image processing

## Abstract

Uncertainty is at the heart of decision-making processes in most real-world applications. Uncertainty can be broadly categorized into two types: aleatory and epistemic. Aleatory uncertainty describes the variability in the physical system where sensors provide information (hard) of a probabilistic type. Epistemic uncertainty appears when the information is incomplete or vague such as judgments or human expert appreciations in linguistic form. Linguistic information (soft) typically introduces a possibilistic type of uncertainty. This paper is concerned with the problem of classification where the available information, concerning the observed features, may be of a probabilistic nature for some features, and of a possibilistic nature for some others. In this configuration, most encountered studies transform one of the two information types into the other form, and then apply either classical Bayesian-based or possibilistic-based decision-making criteria. In this paper, a new hybrid decision-making scheme is proposed for classification when hard and soft information sources are present. A new Possibilistic Maximum Likelihood (PML) criterion is introduced to improve classification rates compared to a classical approach using only information from hard sources. The proposed PML allows to jointly exploit both probabilistic and possibilistic sources within the same probabilistic decision-making framework, without imposing to convert the possibilistic sources into probabilistic ones, and vice versa.

## 1. Introduction

Uncertainty can be categorized into two main kinds [1]: aleatory or randomness uncertainty, aka statistical uncertainty, due to the variability or the natural randomness in a process and epistemic uncertainty, aka systematic uncertainty, which is the scientific uncertainty in the model of the process. It is due to limited data and knowledge. Epistemic uncertainty calls for alternative methods of representation, propagation, and interpretation of uncertainty than just probability. Since the beginning of the 60 s, following fruitful cross-fertilization, a convergence is emerging between physics, engineering, mathematics, and the cognitive sciences to provide new techniques and models that shows a trend of inspiration from human brain mechanism towards a unified theory to represent knowledge, belief and uncertainty [2,3,4,5,6,7,8,9].

Uncertainty is a natural and unavoidable part in real-world applications. When observing a “real-world situation”, decision making is the process of selecting among several alternatives or decisions.

The problem here is to assign a label or a class to measurements or other types of observations (data) from sensors or other sources to which the observations are assumed to belong. This is a typical classification process.

**Figure 1:**
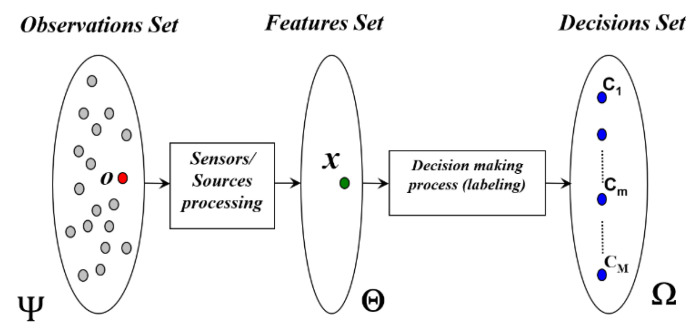
Structure of a multisource classification system (Source: [11]).

As shown in Figure 1, the general classification process can be formulated as follows. An input set of observations ***o*** (o∈Ψ) is “observed” using a sensor (or a set of sensors) delivering a feature vector x∈Θ (Θ is called the features set). This feature vector **x** is then injected into the decision-making system or labelling system in order to recognize the most likely decision (hypothesis, alternative, class) from a given exhaustive set $\Omega =\left\{C_{m}.\;m=1,\ldots ,M\right\}$ of *M* exclusive decisions [10].

The development of both classification algorithms and decision-making criteria are governed by several factors mainly depending on the nature of the feature vector, the nature of the imperfection attached to the observed features as well as the available knowledge characterizing each decision. Several global constraints also drive the conception of the global classification process: the “physical” nature and quality of the measures delivered by the sensors, the categories discrimination capacity of the computed features, the nature and the quality of the available knowledge used for the development of the decision-making system.

However, in much of the literature, the decision-making system is performed by the application of two successive functionalities: the *soft labeling* and the *hard decision (selection)* functionalities. The labeling functionality [12] uses the available a priori knowledge in order to perform a mapping ℓ between the features set Θ and the decisions set Ω (ℓ: Θ⟶Ω). For each feature vector x∈Θ, a *soft decision label* vector $\ell \left(x\right)=\left[\ell_{C_{1}}\left(x\right),\ldots ,\ell_{C_{m}}\left(x\right),\ldots ,\ell_{M}\left(x\right)\;\right]\in {\left[0,1\right]}^{M}$ is determined in the light of the available knowledge where $\ell_{C_{M}}\left(x\right)$ measures the degree of belief or support, that we have in the occurrence of the decision $C_{m}$. For instance, if the available knowledge allows probabilistic computations, the soft decision label vector is given through $\ell_{C_{M}}\left(x\right)=Pr\left\{C_{M}|x\right\}$ [13] where $Pr\left\{C_{M}|x\right\}$ represents the *a posteriori* probability of the decision $C_{M}$ given the observed feature vector x∈Θ [14]. When the available knowledge is expressed in terms of ambiguous information, the possibility theory formalism (developed by L. Zadeh [7] and D. Dubois et al. [15,16,17]) can be used. The soft decision label vector ℓ(x) is then expressed with an *a posteriori* possibility distribution $\pi_{x}$ defined on the decisions set Ω. In this case, $\ell_{C_{m}}\left(x\right)=\pi_{x}\left(C_{m}\right)$ where $\pi_{x}\left(C_{m}\right)$ represents the possibility degree for the decision $C_{m}\;$ to occur, given the observed feature vector x∈Θ.

The second functionality performed by the decision-making process is called the *hard decision* or *the selection functionality*. As the ultimate goal of most classification applications is to select one and only one class (associated with the observations “***o***” for which the feature vector x∈Θ is extracted) out of the classes set Ω, then a mapping has to be applied in order to transform the soft decision label vector ℓ(x) into a hard decision label vector for which one and only one decision is selected. The goal is then to make a choice according to an optimality criterion.

In this paper, we propose a new criterion for decision-making process in classification systems called possibilistic maximum likelihood (PML). This criterion is framed within the possibility theory, but it uses corresponding notions from Bayesian decision-making. The main motivation being the development of PML is for multisource information fusion where an object or a pattern may be observed through several channels and where the available information, concerning the observed features, may be of a probabilistic nature for some features, and of an epistemic nature for some others.

In the presence of both types of information sources, most encountered studies transform one of the two information types into the other form, and then apply either the classical Bayesian or possibilistic decision-making criteria. With the PML decision-making approach, the Bayesian decision-making framework is adopted. The epistemic knowledge is integrated into the decision-making process by defining possibilistic loss values instead of the usually used zero-one loss values. A set of possibilistic loss values is proposed and evaluated in the context of pixel-based image classification where a synthetic scene, composed of several thematic classes, is randomly generated using two types of probabilistic sensors: a Gaussian and a Rayleigh sensor, complemented by an expert type of information source. Results obtained with the proposed PML criterion show that the classification recognition rates approach the optimal case, being, when all the available information is expressed in terms of probabilistic knowledge.

When the sources of information can be modelled by probability theory, the Baysesian approach has sufficient decision-making tools to fuse that information and performs classification. However, in the case where the knowledge available for the decision-making process is ill-defined in the sense that it is totally or partially expressed in terms of ambiguous information representing limitations in feature values, or, encoding linguistic expert’s knowledge about the relationship between the feature values and different potential decisions, new mathematical tools (i.e., PML) need to be developed. This type of available knowledge can be represented as a conditional possibilistic soft decision label vector ℓ(x) defined on the decisions set Ω such that, $\ell_{C_{m}}\left(x\right)=\pi_{x}\left(C_{m}\right)=\pi \left(C_{m}|x\right)$ where $\;\pi \left(C_{m}|x\right)$ represents the possibility degree for the decision *C*_m_ to occur, given the observed feature vector x∈Θ and the underlying observations ***o***.

Possibility theory constitutes the natural framework allowing to tackle this type of information imperfection (called the *epistemic uncertainty* type) when one and only one decision (*hard decision*) must be selected from the exhaustive decisions set Ω, with incomplete, ill-defined or ambiguous available knowledge thus encoded as a possibility distribution over Ω. This paper proposes a joint decision-making criterion which allows to integrate such extra possibilistic knowledge within a probabilistic decision-making framework taking into account both types of information: possibilistic and probabilistic. In spite of the fact that possibility theory deals with uncertainty, which means that a unique but unknown elementary decision is to occur, and the ultimate goal is to determine this decision, there are relatively few studies that tackle that decision-making issue [18,19,20,21,22,23,24,25]. We must however mention the considerable contributions of Dubois and Prade [26] on possibility theory as well as on clarification on the various semantics of fuzzy sets [27,28,29,30]. Denoeux et al. [31,32,33] contributed as well significantly on that topic but they consider epistemic uncertainty as a higher order uncertainty upon probabilistic models such as in imprecise probabilities of Walley [34,35] and fuzzy sets type-2 [36,37,38] which is not being the case in this current paper.

The paper is organized this way. A brief recall of the Bayesian decision-making criteria, and of possibility theory is given in Section 2 and Section 3. Three major possibilistic decision making criteria, i.e., maximum possibility, maximum necessity measure and confidence index maximization, are being detailed in Section 4. The PML criterion is presented in Section 5 followed by its evaluation in Section 6. Paper closes with conclusion in Section 7.

## 2. Hard Decision in the Bayesian Framework

In the Bayesian classification framework, the most widely used hard decision is based on minimizing an overall decision risk function [14]. Assuming o∈Ψ is the pattern for which the feature vector x∈Θ is observed, let $\lambda_{m,n}$ denotes a “predefined” conditional loss or penalty, incurred for deciding that the observed pattern ***o*** is associated with the decision *C_n_*, whereas the true decision (class or category) for ***o*** is $C_{m}\left(n,m\;\epsilon \left\{1,\cdots ,M\right\}\right)$. Therefore, the probabilistic expected loss $R(C_{n}|x)$, also called the *Conditional risk*, associated with the decision $C_{n}$ given the observed feature vector x∈Θ, is given by:

(1) $$R\left(C_{n}|x\right)=E\left\{\lambda_{m,n}\right\}=\overset{M}{\underset{m=1}{\sum }}\lambda_{m,n}Pr\left\{C_{m}|x\right\}$$

where E{·} stands for the mathematical expectation. Bayes decision criterion consists in minimizing the overall risk *R*, also called *Bayes risk*, as defined in (2), by computing the conditional risk for all decisions and then, selecting the decision *C_n_* for which $R(C_{n}|x)$ is minimum:

(2) $$R\left(C_{n}|x\right)=E_{x}\left\{R(C_{n}|x)\;\right\}=\int R(C_{n}|x)Pr\left\{x\right\}dx$$

Therefore, the *minimum-risk Bayes decision criterion* is based on the selection of the decision *C_n_* which gives the smallest risk $R(C_{n}|x)$. This rule can thus be formulated as follows:

(3) $$Decision\left[x\left(p\right)\right]=\arg\underset{n=1,\cdots ,M}{\min}\left(\overset{M}{\underset{m=1}{\sum }}\lambda_{m,n}Pr\left\{C_{m}|x\right\}\right)$$

If $Pr\left\{C_{m}\right\}$ denotes the *a priori* probability of the decision $C_{m}$ and $Pr\left\{x|C_{m}\right\}$, the likelihood function of the measured feature vector ***x***, given the decision *C_m_*, then using Bayes’ rule, the minimum-risk Bayes decision criterion (3) can be rewritten as:

(4) $$Decision\left[x\left(p\right)\right]=\arg\underset{n=1,\cdots ,M}{\min}{\left(\overset{M}{\underset{m=1}{\sum }}Pr\left\{C_{m}\right\}Pr\left\{x|C_{m}\right\}\right)}_{\;}$$

In the two-category decision case, i.e., $\Omega =\left\{C_{1},C_{2}\right\}$, it can be easily shown that the minimum-risk Bayes decision criterion, simply called *Bayes criterion*, can be expressed as in (5):

(5) $$LR=\frac{Pr\left\{x|C_{1}\right\}}{Pr\left\{x|C_{2}\right\}}\;\;\begin{array}{c} \overset{Decision\left[x\left(p\right)\right]=C_{1}}{\overset{︷}{>}}\\ \underset{Decision\left[x\left(p\right)\right]=C_{2}}{\underset{︸}{<}} \end{array}\;\;\underset{\eta }{\underset{︸}{\begin{array}{ccc} \frac{\lambda_{2,2}-\lambda_{2,1\;}}{\lambda_{1,1}-\lambda_{1,2}} & \cdot & \frac{Pr\left\{C_{2}\right\}}{Pr\left\{C_{1}\right\}} \end{array}}}$$

In other words, this decision criterion consists of comparing the likelihood ratio (*LR*) $Pr\left\{x|C_{1}\right\}/Pr\left\{x|C_{2}\right\}\;$ to a threshold *η* independent of the observed feature vector *x*. The binary cost, or zero-one loss, assignment is commonly used in classification problems. This rule, expressed in (6), gives $\lambda_{m,n}$ no cost for a correct decision (when the true pattern class/decision $C_{m\;}$ is identical to the decided class/decision $C_{n\;}$) and a unit cost for a wrong decision (when the true class/decision $C_{m\;}$ is different from the decided class/decision $C_{n\;}$).

(6) $$\lambda_{m,n}=\{\begin{array}{c} \begin{array}{ccc} 0 & \mathrm{if} & C_{m}=C_{n} \end{array}\\ \begin{array}{ccc} 1 & \mathrm{if} & C_{m}\neq C_{n} \end{array} \end{array}$$

It should be noticed that this binary cost assignment considers all errors as equally costly. It also leads to express the conditional risk as:

(7) $$R\left(C_{n}|x\right)=\overset{M}{\underset{m=1}{\sum }}\lambda_{m,n}Pr\left\{C_{m}|x\right\}=1-Pr\left\{C_{n}|x\right\}$$

A decision minimizing the conditional risk $R\left(C_{n}|x\right)$ becomes a decision maximizing the *a posteriori* probability $Pr\left\{C_{n}|x\right\}$. As shown in (8), this version of the Bayes criterion is called the maximum a posteriori criterion (MAP) since it seeks to determine the decision maximizing the *a posteriori* probability value. It is also obvious that this decision process corresponds to the minimum-error decision rule which leads to the best recognition rate that a decision criterion can achieve:

(8) $$Decision_{MAP}\left[x\left(p\right)\right]=\arg\underset{n=1,\cdots M}{\max}Pr\left\{C_{n}|x\right\}$$

When the decisions *a priori* probabilities $Pr\left\{C_{m}\right\}$ and the likelihood functions $Pr\left\{x|C_{m}\right\}\;$ are not available, or simply difficult to obtain, the *Minmax Probabilistic Criterion* (MPC) can be an interesting alternative to the minimum-risk Bayes decision criterion [39]. As expressed in (9), this hard decision criterion consists in selecting the decision that minimizes the maximum decision cost:

(9) $$Decision_{MPC}\left[x\left(p\right)\right]=\arg\;\underset{n=1,\cdots ,M}{\min}\left[\underset{m=1,\cdots ,M}{\max}\left\{\lambda_{m,n}Pr\left\{C_{m}|x\right\}\right\}\right]\;$$

## 3. Brief Review of Possibility Theory

Possibility theory is a relatively new theory devoted to handle uncertainty in the context where the available knowledge is only expressed in an ambiguous form. This theory was first introduced by Zadeh in 1978 as an extension of fuzzy sets and fuzzy logic theory, to express the intrinsic fuzziness of natural languages as well as uncertain information [7]. It is well established that probabilistic reasoning, based on the use of a probability measure, constitutes the optimal approach dealing with uncertainty. In the case where the available knowledge is ambiguous and encoded by a membership function, i.e., a fuzzy set, defined over the decisions set, the possibility theory transforms the membership function into a possibility distribution π. Then the realization of each event (subset of the decisions set) is bounded by a possibilistic interval defined though a possibility, Π, and a necessity, N, measures [16]. The use of these two dual measures in possibility theory makes the main difference compared with the probability theory. Besides, possibility theory is not additive in terms of beliefs combination, and makes sense on ordinal structures [17]. In the following subsections, the basic concepts of a possibility distribution and the dual possibilistic measures (possibility and necessity measures) will be presented. The possibilistic decision rules will be detailed in Section 4. Full details can be found in [11].

### 3.1. Possibility Distribution

Let $\Omega =\left\{C_{1},C_{2},\;\cdots ,C_{M}\right\}$ be a finite and exhaustive set of *M* mutually exclusive elementary decisions (e.g., decisions, thematic classes, hypothesis, etc.). Exclusiveness means that one and only one decision may occur at one time, whereas exhaustiveness states that the occurring decision certainly belongs to Ω. Possibility theory is based on the notion of possibility distribution denoted by π, which maps elementary decisions from Ω to the interval [0, 1], thus encoding “our” state of knowledge or belief, on the possible occurrence of each class $C_{m}\in \Omega$. The value $\pi \left(C_{m}\right)$ represents to what extent it is possible for $C_{m}$ to be the unique occurring decision. In this context, two extreme cases of knowledge are given:

■ Complete knowledge: $\exists !C_{m}\in \Omega ,\;\pi \left(C_{m}\right)=1\;$ and $\pi \left(C_{n}\right)=0,\;\forall C_{n}\in \Omega ,\;C_{n}\neq C_{m}$.
■ Complete ignorance: $\forall C_{m}\in \Omega ,\;\pi \left(C_{m}\right)=1$ (all elements from  Ω are considered as totally possible). π(·) is called a normal possibility distribution if it exists at least one element $C_{m_{0}}$ from Ω such that $\pi \left(C_{m_{0}}\right)=1$.

### 3.2. Possibility and Necessity Measures

Based on the possibility distribution concept, two dual set measures, possibility, Π, and a necessity, N, measures are derived. For every subset (or event) A⊆Ω, these measures are defined by:

(10a) $$\Pi \left(A\right)=\underset{C_{m}\in A}{\max}\left[\pi \left(C_{m}\right)\right]$$

(10b) $$N\left(A\right)=1-\Pi \left(A^{c}\right)=\underset{C_{m}\notin A^{c}}{\min}\left[1-\pi \left(C_{m}\right)\right]$$

where $A^{c}$ denotes the complement of the event *A* (i.e., $A\cup A^{c}=\Omega \;\mathrm{with}\;A\cap A^{c}=\emptyset$).

The possibility measure Π(A) estimates the level of consistency about event *A* occurrence, given the available knowledge encoded by the possibility distribution π. Thus, Π(A)=0 means that *A* is an impossible event while Π(A)=1 means that the event *A* is totally possible. The necessity measure N(*A*) evaluates the level of certainty about event *A* occurrence, involved by possibility distribution π. N(A)=0 means that the certainty about the occurrence of *A* is null. On the contrary, N(A)=1 means that the occurrence of *A* is totally certain. In a classification problem, where each decision $C_{m}$ refers to a given class or category, the case where all events *A* are composed of a single decision $\left(A_{m}=\left\{C_{m}\right\},\;m=1,\;\ldots ,\;M\right)$, is of particular interest. In this case, the possibility Π(·), and the necessity N(·), measures are reduced to:

(11a) $$\Pi \left(A_{m}\right)=\Pi \left(\left\{C_{m}\right\}\right)=\pi \left(C_{m}\right)$$

(11b) $$N\left(A_{m}\right)=N\left(\left\{C_{m}\right\}\right)=1-\Pi \left({\left\{C_{m}\right\}}^{c}\right)=1-\underset{n\neq m}{\max}\pi \left(C_{n}\right)$$

## 4. Decision-Making in the Possibility Theory Framework

In this section, we will investigate existing possibilistic decision-making rules. Two families of rules can be distinguished: rules based on the direct use of the information encapsulated in the possibility distribution, and rules based on the use of uncertainty measures associated with this possibility distribution. Let $\Omega =\left\{C_{1},C_{2},\;\cdots ,C_{M}\right\}$ be a finite and exhaustive set of *M* mutually exclusive elementary decisions. Given an observed pattern o∈Ψ for which the feature vector x∈Θ is observed, let $\pi_{x}\left(C_{m}\right)$ denotes the *a posteriori* possibility distribution $\pi (C_{m}|x)$ defined on Ω. The possibility, $\Pi_{x}\left(\left\{C_{m}\right\}\right)$, and necessity, $N_{x}\left(\left\{C_{m}\right\}\right)$, measures are obtained as expressed in Equation (11), using the possibility distribution $\pi_{x}\left(C_{m}\right)$.

### 4.1. Decision Rule Based on the Maximum of Possibility

The decision rule based on the maximum of possibility is certainly the most widely used in possibilistic classification—decision-making applications. Indeed, as shown in (12), this rule is based on the selection of the elementary decision $C_{m_{0}}\in \Omega$ having the highest possibility degree of occurrence $\Pi_{x}\left(\left\{C_{m_{0}}\right\}\right)$:

(12) $$Decision\;\left[x\left(p\right)\right]=\;C_{m_{0}}\;\mathrm{if}\;\mathrm{and}\;\mathrm{only}\;\mathrm{if}\;m_{0\;}=\arg\underset{n=1,\cdots M}{\max}\Pi_{x}\left(\left\{C_{m}\right\}\right)$$

A “first” mathematical justification of this “intuitive” possibilistic decision-making rule can be derived from the *Minmax Probabilistic Criterion* (MPC), Equation (9), using a binary cost assignment rule. Indeed, ‘converting’ the *a posteriori* possibility distributions $\pi_{x}\left(\cdot \right)\;$ into *a posteriori* probability distributions Pr{·|x} is assumed to respect the three following constraints [30]: (a) the consistency principle, (b) the preference ordering preservation, and (c) the least commitment principle. The preference ordering preservation, on which we focus the attention here, means that if decision $C_{m_{1}}$ is preferred to decision $C_{m_{2}}$, i.e., $\pi_{x}\left(C_{m_{1}}\right)>\pi_{x}\left(C_{m_{2}}\right)$, then the *a posteriori* probability distribution Pr{·|x} obtained from $\pi_{x}\left(\cdot \right)\;$ should satisfy $Pr\left\{C_{m_{1}}|x\right\}>Pr\left\{C_{m_{2}}|x\right\}$. Equation (13) sums up this preference ordering preservation constraint:

(13) $$\pi_{x}\left(C_{m_{1}}\right)>\pi_{x}\left(C_{m_{2}}\right)⟺Pr\left\{C_{m_{1}}|x\right\}>Pr\left\{C_{m_{2}}|x\right\}$$

Therefore, selecting the decision maximizing the *a posteriori* probability or selecting the decision maximizing the *a posteriori* possibility decision is identical: using the MPC associated with the binary cost assignment rule or using the maximum possibility decision rule led to an identical result as expressed in (14).

(14) $$Decision\;\left[x\left(p\right)\right]=\;C_{m_{0}}\;\mathrm{iff}\;m_{0\;}=\arg\underset{m=1,\cdots M}{\max}Pr\left\{C_{m}|x\right\}=\underset{m=1,\cdots M}{\max}\pi_{x}\left(C_{m}\right)$$

This decision-making criterion is called the *Naive Bayes style* possibilistic criterion Refs. [40,41,42] and most ongoing efforts are oriented into the computation of the *a posteriori* possibility values using numerical data [43]. An extensive study of properties and equivalence between possibilistic and probability approaches is presented in [20]. Notice that this decision rule, strongly inspired from probabilistic decision reasoning, does not provide a hard decision mechanism when several elementary decisions have the same maximum possibility measure.

### 4.2. Decision Rule Based on Maximizing the Necessity Measure

It is worthwhile to notice that the *a posteriori* measures of possibility $\Pi_{x}$ and necessity $N_{x}$ coming from a normal *a posteriori* possibility distribution $\pi_{x}\left(\cdot \right)$, constitute a bracketing for the *a posteriori* probability distribution Pr{·|x} [17]:

(15) $$N_{x}\left(\left\{C_{m}\right\}\right)=1-\underset{\begin{array}{c} n\neq m\\ n=1.\cdots ,M \end{array}}{\max}\pi_{x}\left(C_{n}\right)\leq Pr\left\{C_{m}|x\right\}\leq \Pi_{x}\left(\left\{C_{m}\right\}\right)=\pi_{x}\left(C_{m}\right)$$

Therefore, the maximum possibility decision criterion can be considered as an optimistic decision criterion as it maximizes the upper bound of the *a posteriori* probability distribution. On the contrary, a pessimistic decision criterion based on maximizing the *a posteriori* necessity measure can be considered as a maximization of the lower bound of the *a posteriori* probability distribution. Equation (16) expresses this pessimistic decision criterion:

(16) $$Decision\;\left[x\left(p\right)\right]=\;C_{m_{0}}\;\mathrm{iff}\;m_{0\;}=\arg\underset{n=1,\cdots M}{\max}\left[N_{x}\left(\left\{C_{m}\right\}\right)\right]$$

The question that we must raise concerns the “links” between the optimistic and the pessimistic decision criteria. Let us consider the *a posteriori* possibility distribution $\pi_{x}\left(\cdot \right)$ for which $C_{m_{1}}\left(\mathrm{resp}.\;C_{m_{2}}\right)$ is the “winning decision” obtained using the maximum possibility (resp. necessity measure) decision criteria as given in (17):

(17) $$\pi_{x}\left(C_{m_{1}}\right)=\underset{m}{\max}\pi_{x}\left(C_{m}\right)and\;N_{x}\left(\left\{C_{m_{2}}\right\}\right)=\underset{m}{\max}N_{x}\left(\left\{C_{m}\right\}\right)$$

The following important question can be formulated as follows: “Is the winning decision $C_{m_{1}}$ (according to the maximum possibility criterion) is the same as the winning decision $C_{m_{2}}$ according to maximum necessity measure criterion?”

First, notice that if several elementary decisions share the same maximum possibility value $\upsilon =\pi_{x}\left(C_{m_{1}}\right)$, then, the necessity measure becomes a useless decision criterion since:

$$N_{x}\left(\left\{C_{m}\right\}\right)=1-\underset{k\neq m}{\max}\pi_{x}\left(C_{k}\right)=1-\upsilon \;\mathrm{for}\;\mathrm{all}\;\mathrm{the}\;\mathrm{elementary}\;\mathrm{decisions}.$$

Now, suppose that only one decision $C_{m_{1}}$ assumes the maximum possibility value $\upsilon =\pi_{x}\left(C_{m_{1}}\right)$, it is important to raise the question whether the decision $C_{m_{1}}$ will (or will not) be the decision assuming the maximum necessity measure value. Let us note *v*’, the possibility value for the “second best” decision according to the possibility value criterion. As $C_{m_{1}}$ is the unique decision having the maximum possibility value υ, we have $\upsilon^{\prime }<\upsilon$. Therefore, as shown in (18), the necessity measure value $N_{x}\left(\left\{C_{m}\right\}\right)$ only gets maximum for the decision $C_{m_{1}}\;$ since $1-\upsilon^{\prime }>1-\upsilon$.

(18) $$N_{x}\left(\left\{C_{m}\right\}\right)=1-\underset{k\neq m}{\max}\pi_{x}\left(C_{k}\right)=\{\begin{array}{c} \begin{array}{cc} 1-\upsilon^{\prime }\;\;\;\mathrm{if} & m= \end{array}\;m_{1}\;\\ \begin{array}{cc} 1-\upsilon \;\;\;\mathrm{if} & m\neq \end{array}\;m_{1} \end{array}$$

**Figure 2:**
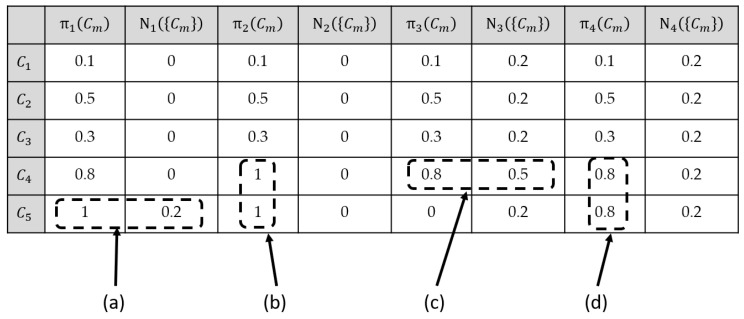
Comparative example of the maximum possibility and maximum necessity measures decision criteria using four *a posteriori* possibility distributions.

As a conclusion, when the maximum necessity measure criterion is useful for application (i.e., only one elementary decision assumes the maximum possibility value), then, both decision criteria (maximum possibility and maximum necessity) produce the same winning decision. In order to illustrate the difference between the maximum possibility and the maximum necessity measure criteria, Figure 2 presents an illustrative example.

In Figure 2 example, four different *a posteriori* possibility distributions $\pi_{1},\;\pi_{2},\pi_{3},\pi_{4}$, all defined on a five elementary decisions set $\Omega =\left\{C_{1},C_{2},C_{3},C_{4},C_{5}\right\}$ are considered. The necessity measures $N_{k}\left(\left\{C_{m}\right\}\right)$ have been computed from the corresponding possibility distribution $\pi_{k}$. The underlined values indicate which decisions result from the maximum possibility decision criterion as well as the maximum necessity measure decision criterion, for the four possibility distributions $\pi_{k}$. Note that the necessity measure assumes at most two values whatever the considered possibility distribution. When the *a posteriori* possibility distribution has one and only one decision having the highest possibility degree, then both decision rules produce the same winning decision. This is the case of the normal possibility distribution $\pi_{1}$ as well as the subnormal possibility distribution $\pi_{3}$, indicated as cases (a) and (c) in Figure 2.

When several elementary decisions share the same highest possibility degree, then the maximum possibility decision criterion can randomly select one of these potential winning decisions. In this case, the maximum necessity measure decision criterion will affect a single necessity measure degree to all elementary decisions from Ω, and thus, it will be impossible to select any of the potential winning decisions. This behavior can be observed with a normal possibility distribution $\pi_{2}$ as well as with a subnormal possibility distribution (like $\pi_{4}$), cases (b) and (d) in Figure 2. This example clearly shows the weakness of the decisional capacity of the maximum necessity measure decision criterion when compared to the maximum possibility decision criterion.

### 4.3. Decision Rule Based on Maximizing the Confidence Index

Other possibilistic decision rules based on the use of uncertainty measures are also encountered in literature. The most frequently used criterion (proposed by Kikuchi et al. [44]) is based on the maximization of the confidence index *Ind* defined as a combination of the possibility and the necessity measures for each event A⊆Ω, given a possibility distribution π(·):

$$Ind:\;2^{\Omega }⟶\left[-1,\;+1\right]$$

(19) A⟶Ind(A)=Π(A)+N(A)−1, ∀A⊆Ω

where $2^{\Omega }$ denotes the power set of Ω, i.e., the set of all subsets from Ω.

For an event *A*, this index ranges from −1 to +1:

- *Ind*(*A*) = −1, iff Π(A)=N(A)=0 (the occurrence of *A* is totally impossible and uncertain);
- *Ind*(*A*) = +1, iff Π(A)=N(A)=1 (the occurrence of *A* is totally possible and certain).

Restricting the application of this measure to events $A_{m}$ having only one decision $A_{m}=\left\{C_{m}\right\}$ shows that *Ind*($A_{m}$) measures the difference between the possibility measure of the event $A_{m}$ (which is identical to the possibility degree of the decision $C_{m}$) and the highest possibility degree of all decisions contained in ${\left(A_{m}\right)}^{c}$ (the complement of $A_{m}$ in Ω):

(20) $$Ind\left(A_{m}\right)=\;\Pi \left(A_{m}\right)+N\left(A_{m}\right)-1=\pi \left(C_{m}\right)-\underset{m\neq n}{\max}\pi \left(C_{n}\right)$$

**Figure 3:**
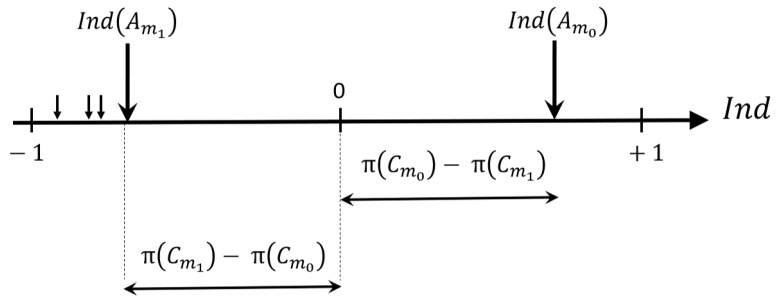
Confidence indices associated with different decisions ($A_{m_{0}}$: event having the highest possibility degree, $A_{m_{1}}$: event with the second highest possibility degree). (Source: [11]).

Therefore, if $A_{m_{0}}=\left\{C_{m_{0}}\right\}$ is the only event having the highest possibility measure value $\pi \left(C_{m_{0}}\right)$, then, $A_{m_{0}}$ will be the unique event having a positive confidence index value, whereas all other events will have negative values, as illustrated in Figure 3 where we assume $\pi \left(C_{m_{0}}\right)>\pi \left(C_{m}\right),\;\forall m\neq m_{0}$, and, $C_{m_{1}}$ refers to the decision having the second highest possibility degree.

In a classification decision-making problem, the decision criterion associated with this index can be formulated as follows:

(21) $$Decision_{Ind}\;\left[x\left(p\right)\right]=\;A_{m_{0}}\;\mathrm{iff}\;Ind\left(A_{m_{0}}\right)=\underset{m=1,\cdots M}{\max}\left[Ind\left(A_{m}\right)\right]$$

The main difference between the maximum possibility and the maximum confidence index decision criteria lies in the fact that the maximum possibility decision criterion is only based on the maximum possibility degree whereas the maximum confidence index decision criterion is based on the difference between the two highest possibility degrees associated with the elementary decisions. As already mentioned, it is important to notice that the event $A_{m_{0}}=\left\{C_{m_{0}}\right\}$ having the highest possibilistic value, will be the unique event producing a positive confidence index measuring the difference with the second highest possibility degree. All other events $A_{m}=\left\{C_{m}\right\},\;\forall m\neq m_{0}$, will produce negative confidence indices.

When several decisions share the same highest possibility degree, their confidence index (the highest one) will be null. This shows the real capacity of this uncertainty measure for the decision-making process. However, this criterion brings the same resulting decisions as the two former ones.

## 5. Possibilistic Maximum Likelihood (PML) Decision Criterion

In the formulation of the Bayesian classification approach, all information sources are assumed to have probabilistic uncertainty where the available knowledge describing this uncertainty is expressed, estimated or evaluated in terms of probability distributions. In the possibilistic classification framework, the information sources are assumed to suffer from possibilistic (or epistemic) uncertainty where the available knowledge describing this uncertainty is expressed in terms of possibility distributions. In this section, the Bayesian pattern recognition framework is generalized in order to integrate both probabilistic and epistemic sources of knowledge. A joint probabilistic—possibilistic decision criterion called *Possibilistic Maximum Likelihood* (PML) is proposed to handle both types of uncertainties.

### 5.1. Sources with Probabilistic and Possibilistic Types of Uncertainties

In some situations, an object from the observation space is observed through several feature sets. This is the case, for instance, in multi-sensor environment for classification applications. In such situations, the information available for the description of the feature vectors may be of different natures: probabilistic, epistemic, etc. Yager [24,45,46] addresses the same sort of problems: multi-source uncertain information fusion in the case when the information can be both from hard sensors of a probabilistic type and from soft knowledge-expert linguistic source of a possibilistic type. He uses t-norms (‘*and*’ operations) to combine possibility and probability measures. As will be explained below, Yager’s product of possibilities and probabilities coincides with our ‘decision variables’ optimized through the proposed PML approach.

**Figure 4:**
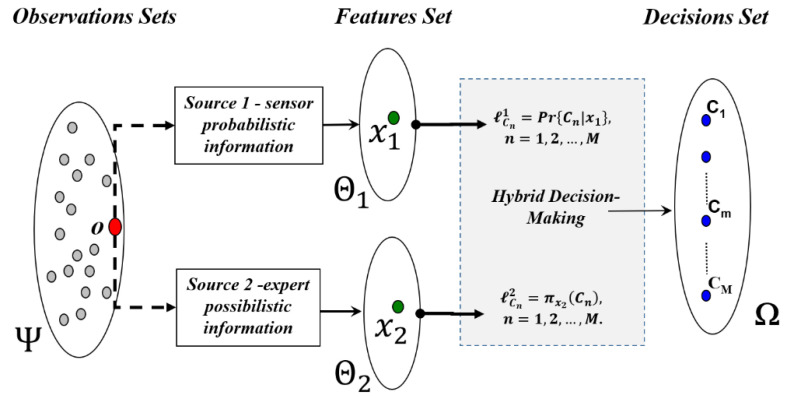
Multi-source information context for pattern classification.

Let us consider the example illustrated in Figure 4, where each pattern ***o*** (from the patterns set Ψ**)** is “observed” through two channels. Source 1 (resp. source 2) measures a sub-feature vector $x_{1}\in \Theta_{1}\left(\mathrm{resp}.x_{2}\in \Theta_{2}\right)$. Therefore, the resulting feature vector x(o) is obtained as the concatenation of the two sub-feature vectors: $x\left(o\right)=\left[x_{1}\;x_{2}\right]$. In this configuration, the available information in the sub-feature vector ***x*_1_** (resp. ***x*_2_**) undergoes probabilistic (resp. epistemic) uncertainties and is encoded as an *a posteriori* probability soft decision label vector $\ell_{C_{n}}^{1}=Pr\left\{C_{n}|x_{1}\right\},\;n=1,2,\;\ldots ,\;M\;$ (resp. *a posteriori* possibility soft decision label vector $\ell_{C_{n}}^{2}=\pi_{x_{2}}\left(C_{n}\right),\;n=1,2,\;\ldots ,\;M)$.

As an example, in a remote sensing system, *Source 1* may be considered as a multispectral imaging system, where all potential *a posteriori probability distributions*, $Pr\left\{C_{n}|x_{1}\right\},\;n=1,2,\;\ldots ,\;M$, are assumed to be known and well established. The second sensor, *Source 2*, could be a radar imaging system where the available information concerning the different thematic classes is expressed by an expert using ambiguous linguistic variables like: “the thematic class $C_{n\;}$ is observed as “*Bright*”, “*Slightly Dark*”, etc. in the sub-feature set $\Theta_{2}$”. Each linguistic variable can be used to generate an *a posteriori possibility distribution* associated with each thematic class $\pi_{x_{2}}\left(C_{n}\right),\;n=1,2,\;\ldots ,\;M$.

### 5.2. Possibilistic Maximum Likelihood (PML) Decision Criterion: A New Hybrid Criterion

In the Bayesian decision framework, detailed in previous sections, the binary cost assignment approach suffers from two constraints. On one hand, all errors are considered as equally costly: the penalty (or cost) of misclassifying an observed pattern ***o*** as being associated with a decision $C_{n}$ whereas the true decision for “***o***” is $C_{m}$ is the same (unit loss). This situation does not reflect real applications constraints. For instance, deciding that an examined patient is healthy whereas he suffers a cancer is much more serious than the other way around. On the other hand, the loss function values $\lambda_{m,n},\;\forall m,n\in \left\{1,2,\ldots ,M\right\}$ are static (or, predefined) and do not depend on the feature vectors of the observed patterns. The possibilistic maximum likelihood (PML) criterion, proposed in this paper, is based on the use of the epistemic source of information (the *a posteriori* possibility distribution, defined on the sub-feature space $\Theta_{2}$) in order to define possibilistic loss values and to inject, afterwards, these values into the Bayesian decision criterion.

Assume that, for each object o∈Ψ, the observed feature vector is given by $x\left(o\right)=\left[x_{1}\;x_{2}\right]\in \Theta_{1}\times \Theta_{2}$, and denote $Pr\left\{\cdot |x_{1}\right\}\;(\mathrm{resp}.\pi_{x_{2}}\left(\cdot \right)$) as encoding the *a posteriori* probability (resp. possibility) soft decision label vectors defined over the sub-feature set $\Theta_{1}$ (resp. $\Theta_{2}$). The proposed PML criterion relies on the use of loss values $\lambda_{m,n}$ ranging from −1 (i.e., no loss) to +1 (i.e., maximum loss), and $\lambda_{m,n}$ refers to the risk of choosing $C_{n}$ whereas the real decision for the considered pattern is $C_{m}$. Depending on the epistemic information available through Source 2, the proposed loss values are given by:

(22) $$\lambda_{m,n}=\{\begin{array}{c} \begin{array}{cc} \begin{array}{cc} \underset{k\neq m}{\max}\pi_{x_{2}}\left(C_{k}\right)\;\;\;= & \Pi_{x_{2}}\left({\left\{C_{m}\right\}}^{c}\right) \end{array} & \forall m\neq n \end{array}\\ \begin{array}{cc} \underset{k\neq m}{\max}\pi_{x_{2}}\left(C_{k}\right)-\pi_{x_{2}}\left(C_{m}\right)\;=-Ind_{x_{2}}\left(\left\{C_{m}\right\}\right) & \mathrm{if}\;\;\;m=n \end{array}\; \end{array}$$

In the case of a wrong decision, the decision penalty values, i.e., $\lambda_{m,n}$ where ≠m, are considered as positive loss values ranging in the interval: 0≤ $\lambda_{m,n}$ = $\underset{k\neq m}{\max}\pi_{x_{2}}\left(C_{k}\right)\leq 1.$ Thus, the wrong decision unit cost in the framework of binary-cost assignment, is “softened”, in this possibilistic approach, and assumes its maximum value, i.e., unit cost, only when the wrong decision $C_{n}\;$ has a total possibility degree of occurrence.

When a correct decision $C_{m}$ is selected, the zero-loss value (used by the binary cost assignment approach) is substituted by $\lambda_{m,n}=\underset{k\neq m}{\max}\pi_{x_{2}}\left(C_{k}\right)-\pi_{x_{2}}\left(C_{m}\right)=-Ind_{x_{2}}\left(\left\{C_{m}\right\}\right)$. If the occurrence possibility degree $\pi_{x_{2}}\left(C_{m}\right)$ of the true decision $C_{m}$, is the highest degree $\pi_{x_{2}}\left(C_{m}\right)>\underset{k}{\max}\pi_{x_{2}}\left(C_{k}\right)$, then the resulting loss value $\lambda_{m,n}$ becomes negative. The smallest penalty value is reached, i.e., $\lambda_{m,n}=-1$, when $\pi_{x_{2}}\left(C_{m}\right)=1$ (i.e., true decision $C_{m}$ has a total possibility degree of occurrence), with a null possibility degree of occurrence for all the remaining decisions (leading to $\underset{m\neq k}{\max}\pi_{x_{2}}\left(C_{k}\right)=0$). Two special cases are present:

(1) If the true decision $C_{m}$ shares the same maximum possibility value with, at least one different wrong decision $C_{m}$, then, the correct decision $C_{m}$ loss value becomes null $\lambda_{m,n}=\underset{k\neq m}{\max}\pi_{x_{2}}\left(C_{k}\right)-\pi_{x_{2}}\left(C_{m}\right)\;=0$;
(2) If the true decision $C_{m}$ does not produce the maximum occurrence possibility degree, i.e.,$\;\pi_{x_{2}}\left(C_{m}\right)<\underset{k\neq m}{\max}\pi_{x_{2}}\left(C_{k}\right)$, then the loss value $\lambda_{m,n}$ is positive and will increase the conditional risk, associated with the true decision $C_{m}$.

Using the proposed possibilistic loss values, the conditional risk $R\left(C_{k}|x_{1}\right)$ of choosing decision *C_k_* can thus be computed as follows:

(23) $$R\left(C_{k}|x\right)\;=-Ind_{x_{2}}\left(\left\{C_{k}\right\}\right)\cdot Pr\left\{C_{k}|x_{1}\right\}+\overset{M}{\underset{\begin{array}{c} i=1\\ i\neq k \end{array}}{\sum }}\Pi \left({\left\{C_{m}\right\}}^{c}\right)\cdot \;Pr\left\{C_{i}|x_{1}\right\}$$

As already mentioned, Bayes decision criterion computes the conditional risk for all decisions, then, selects the decision $C_{n}$ for which $R\left(C_{n}|x\right)$ is minimum. Based on Equation (23), and to select the minimum conditional risk decision, the comparison of conditional risks related to two decisions $C_{k}$ and $C_{p}$, can be straightforward performed leading to:

(24) $$R\left(C_{k}|x_{1}\right)\leq \;R\left(C_{p}|x_{1}\right)\Leftrightarrow \pi_{x_{2}}\left(C_{k}\right)\cdot Pr\left\{C_{k}|x_{1}\right\}\geq \pi_{x_{2}}\left(C_{k}\right)\cdot \;Pr\left\{C_{p}|x_{1}\right\}$$

Therefore, the application of the PML criterion, for the selection of the minimum conditional risk decision (out of *M* potential elementary decisions) can be simply formulated by the following decision rule:

(25) $$Decision\;\left[x\left(p\right)\right]=\arg\underset{n=1,\cdots M}{\max}\pi_{x_{2}}\left(C_{n}\right)\cdot Pr\left\{C_{n}|x_{1}\right\}$$

This “intuitive” decision criterion allows the joint use both probabilistic and epistemic sources of information in the very same Bayesian minimum risk framework. As an example, the application of the proposed possibilistic loss values in the two-class decision case, where $\Omega =\left\{C_{1},C_{2}\right\}$, leads to the following loss matrix [λ]:

(26) $$\left[\lambda \right]=\left[\begin{array}{cc} \lambda_{1,1} & \lambda_{1,2}\\ \lambda_{2,1} & \lambda_{2,2} \end{array}\right]=\left[\begin{array}{cc} \pi_{x_{2}}\left(C_{2}\right)-\pi_{x_{2}}\left(C_{1}\right) & \pi_{x_{2}}\left(C_{2}\right)\\ \pi_{x_{2}}\left(C_{1}\right) & \pi_{x_{2}}\left(C_{1}\right)-\pi_{x_{2}}\left(C_{2}\right) \end{array}\right]$$

The use of this loss matrix [λ] into the minimum-risk Bayes decision approach (as defined in (5)), leads to express the PML decision as follows:

(27) $$\frac{Pr\left\{x_{1}|C_{1}\right\}}{Pr\left\{x_{1}|C_{2}\right\}}\;\;\begin{array}{c} \overset{Decision\left[x\left(p\right)\right]=C_{1}}{\overset{︷}{>}}\\ \underset{Decision\left[x\left(p\right)\right]=C_{2}}{\underset{︸}{<}} \end{array}\;\;\begin{array}{ccc} \frac{\pi_{x_{2}}\left(C_{2}\right)}{\pi_{x_{2}}\left(C_{1}\right)} & \cdot & \frac{Pr\left\{C_{2}\right\}}{Pr\left\{C_{1}\right\}} \end{array}$$

Notice that when the proposed possibilistic loss values are considered, then the PML induces a “weighting adjustment” of the *a priori* probabilities where the weighting factors are simply the *a posteriori* possibility degrees issued from the possibilistic Source 2. In the case of equal *a priori* probabilities, $Pr\left\{C_{2}\right\}=Pr\left\{C_{1}\right\}$, this decision criterion turns to an intuitive form using jointly probabilistic and epistemic sources of information, in the Bayesian minimum risk framework as shown by:

(28) $$\pi_{x_{2}}\left(C_{1}\right)\cdot Pr\left\{x_{1}|C_{1}\right\}\;\;\begin{array}{c} \overset{Decision\left[x\left(p\right)\right]=C_{1}}{\overset{︷}{>}}\\ \underset{Decision\left[x\left(p\right)\right]=C_{2}}{\underset{︸}{<}} \end{array}\;\pi_{x_{2}}\left(C_{2}\right)\cdot Pr\left\{x_{1}|C_{2}\right\}$$

It is worthwhile to notice that when the two following conditions prevail:

- when the available probabilistic information (issued from source 1) is non-informative; and,
- when the only meaningful and available information is reduced to the epistemic expert information on the sub-feature vector issued from source 2; then, the proposed PML criterion is simply reduced to the maximum possibility decision criterion:
  (29) $$\pi_{x_{2}}\left(C_{1}\right)\;\;\begin{array}{c} \overset{Decision\left[x\left(p\right)\right]=C_{1}}{\overset{︷}{>}}\\ \underset{Decision\left[x\left(p\right)\right]=C_{2}}{\underset{︸}{<}} \end{array}\;\pi_{x_{2}}\left(C_{2}\right)\;$$

This raises a fundamental interpretation of the maximum possibility decision criterion as being a very special case of the possibilistic Bayesian decision making process under the total ignorance assumption of the probabilistic source of information.

### 5.3. PML Decision Criterion Behavior

Let $S_{1}$ denotes a probabilistic source of information measuring a sub-feature vector $x_{1}\in \Theta_{1}$ and attributing to each elementary decision $C_{m},\;m=1,2,\ldots ,M$, an *a posteriori* probability soft decision label $Pr\left\{C_{m}|x_{1}\right\}$. Under the assumption of equal *a priori* probabilities and using the binary-cost assignment, the application of the maximum *a posteriori* criterion (MAP), Equation (8), turns to be the “optimal” criterion ensuring the minimum-error decision rate.

Assume that an additional possibilistic source of information, $S_{2}$, (measuring a sub-feature vector $x_{2}\in \Theta_{2})$ is available, see Figure 4. Based on the use of the sub-feature vector $x_{2}\in \Theta_{2}$, $S_{2}$ attributes to each elementary decision, $C_{m}$, an *a posteriori* possibility soft decision label $\pi_{x_{2}}\left(C_{m}\right),\;m=1,2,\ldots ,M$. To obtain a hard decision, the application of the *maximum of possibility* decision criterion, Equation (12), is considered.

In the previous section, we have proposed the possibilistic maximum likelihood, PML, decision criterion, Equation (25), as a hybrid decision criterion allowing the coupled use of both sources of information, $S_{1}$ and $S_{2}$, by considering the possibilistic information issued from $S_{2}$, i.e., $\pi_{x_{2}}\left(C_{m}\right),\;m=1,2,\ldots ,M$, for the definition of the loss values in the framework of the minimum-risk Bayes decision criterion (instead of the use of the binary-cost assignment approach). In this section, we will briefly discuss, from a descriptive point of view through an illustrative example, the “decisional behavior” of the PML criterion when compared to the decisions obtained with the “individual” application of the MAP and the *maximum of possibility* decision criteria.

First, it is worthwhile to notice that the “decision variable” to be maximized by the PML criterion is simply the direct product $\upsilon \left(C_{m}\right)=\pi_{x_{2}}\left(C_{m}\right)\cdot Pr\left\{C_{m}|x_{1}\right\},\;m=1,2,\ldots ,M$, which is a T-norm fusion operator (considering both probabilistic and possibilistic information as two “similar” measures of the degree of truthfulness related to the occurrence of different elementary decisions, see also p.101 of Yager [24]). This also means that both sources of information, $S_{1}$ and $S_{2}$, are considered as having the same informative level. It is also important to notice that the PML criterion, as a decision fusion operator merging decisional information from both sources, $S_{1}$ and $S_{2}$, constitutes a coherent decision fusion criterion in the sense that:

- when both sources $S_{1}$ and $S_{2}$ are in full agreement (i.e., leading to the same decision $C_{m_{0}}$), then, the decision obtained by the application of the PML criterion will be the same as $C_{m_{0}}$;
- when one of the two sources $S_{1}$ and $S_{2}$ suffers from total ignorance (i.e., producing equal *a posteriori* probabilities, for $S_{1}$ and equal *a posteriori* possibilities, for $S_{2}$), then the PML criterion will “duplicate” the same elementary decision as the one proposed by the remaining reliable source of information;
- when the two sources $S_{1}$ and $S_{2}$ lack decisional agreement, then, the decision obtained by the application of the PML criterion will be the most “plausible” elementary decision that may be different from individual decisions resulting from the MAP (resp. maximum possibility) criterion using the sub-feature vector $x_{1}\in \Theta_{1}$ (resp. sub-feature vector $x_{2}\in \Theta_{2}$).

This decision fusion coherence is illustrated through the examples given in Table 1. The decisions set is formed by five elementary decisions, i.e., $\Omega =\left\{C_{1},C_{2},C_{3},C_{4},C_{5}\right\}$, and we assume that, given the observed feature $x_{1}\in \Theta_{1}$, the probabilistic source $S_{1}$ produces the following *a posteriori* probability distribution: $Pr\left\{\cdot |x_{1}\right\}$ = [0.1 0.4 0.1 0.3 0.1]. Each example fits in one sub-array which presents $S_{1}$ and $S_{2}$ specific configuration ($Pr\left\{\cdot |x_{1}\right\}$, $\pi_{x_{2}}\left(\cdot \right)$ and $\upsilon =Pr\left\{\cdot |x_{1}\right\}\cdot \pi_{x_{2}}\left(\cdot \right))$ with the resulting decision for each decision parameter. The cases presented in Table 1 are explained as the following:

*Case 1*: when both sources $S_{1}$ and $S_{2}$ agree, with a winning decision $C_{2}$, the PML criterion maintains this agreement and obtains the same decision, $C_{2}$.
*Cases 2 and 3*: it shows that when one of the two sources presents a total ignorance, then the PML criterion “duplicates” the same elementary decision as the one offered by the remaining reliable source of information.
*Cases 4, 5 and 6*: when sources $S_{1}$ and $S_{2}$ lack agreement (i.e., dissonant sources), then, the resulting decision obtained through the application of the PML criterion is the most reasonable decision. That may not necessarily be one of the winning decisions offered by the two sources (this is specifically shown in case 6).

## 6. Experimental and Validation Results

In this section, the proposed PML decision-making criterion is evaluated in a pixel-based image classification context. A synthetic scene composed of five thematic classes $\Omega =\left\{C_{1},C_{2},C_{3},C_{4},C_{5}\right\}$ is assumed to be observed through two independent imaging sensors. Sensor $S_{1}$ (resp. sensor $S_{2}$) provides an image $I_{1}$ (resp. $I_{2}$) of the simulated scene. The two considered sensors are assumed to be statistically independent. Without loss of generalization, pixels from both images $I_{1}$ and $I_{2}$ are assumed to have the same spatial resolution, thus, they represent the same observed spatial cell or *object **o***. The value of the pixel $I_{1}\left(i,j\right)$ (resp. $I_{2}\left(i,j\right)$) provides the observed feature $x_{1}\left(\mathrm{resp}.x_{2}\right)$ delivered by the first (resp. second) sensor. According to sensors characteristics, the measured feature $x_{1}\left(\mathrm{resp}.x_{2}\right)$ follows a Gaussian $N\left(m_{C},\sigma_{C}^{2}\right)$ (resp. Rayleigh $ℛ\left(\sigma_{C}^{2}\right)$) probability distribution with related parameters $m_{C},\;\sigma_{C}^{2}\;$ depending on the thematic class “C” of the observed object.

**Figure 5:**
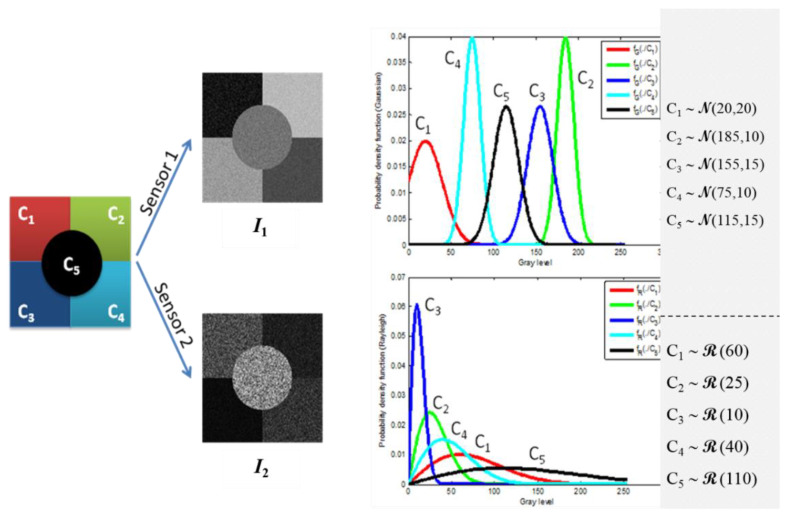
Two-sensors simulated images representing a scene of five thematic classes. Pixels from $I_{1}$ (resp. $I_{2}$) are generated using Gaussian (resp. Rayleigh) density functions.

**Figure 6:**
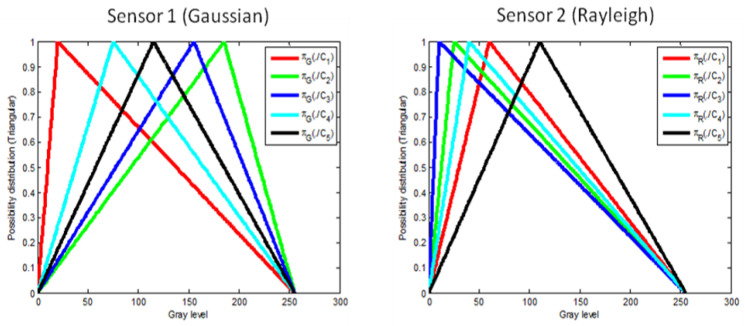
Triangular-shaped possibility distributions encoding expert’s knowledge, for the five thematic classes and the two sensors.

Figure 5 depicts the experimental simulated images $I_{1}$ (resp. $I_{2}$) assumed to be delivered at the output of the two sensors. Figure 6 shows the possibility distributions encoding expert’s information, for the five thematic classes. Parameter values considered for each thematic class are given in the same figure. This configuration of classes’ parameters is considered as a reasonable configuration that may be encountered when real data is observed. Nevertheless, other configurations have been generated and the obtained results are in full accordance with those obtained by the considered configuration.

In addition to the previously mentioned probabilistic information, we assume that each thematic class is described, by an expert, using the “simplest” linguistic variable “*Close to* $v_{S,C_{k}}$” where $v_{S,C_{k}}$ denotes the thematic class $C_{k}$ feature mean value, observed through sensor $S_{s}$. Therefore, the only information given by the expert is $v_{1,C_{k}}=m_{C_{K}}$ for sensor $S_{1}$ (underlying Gaussian distributions) and $v_{2,C_{k}}=\sigma_{C_{K}}\sqrt{\pi /2}$ for sensor $S_{2}$ (underlying Rayleigh distributions). For each sensor $S_{s}$ and thematic class $C_{k}$, a standard triangular possibility distribution is considered to encode this epistemic knowledge with the summit positioned at the mean value $v_{S,C_{k}}$ and the support covering the whole range of the features set. It is clearly seen that the possibility distributions (considered as encoding the expert’s knowledge), represent a weak knowledge which is *less* informative than the initial, or even estimated, probabilistic density functions.

To evaluate the efficiency of the proposed possibilistic maximum likelihood decision making criterion, the adopted procedure consists, first, on the random generation of 1000 statistical realizations of the two synthetic Gaussian and Rayleigh images (with the five considered thematic classes) representing the analyzed scene. Second, the following average pixel-based recognition rates are evaluated:

- $\tau \left(Pr_{C_{m}}^{G}\right)$: Minimum-risk Bayes average pixel-based decision recognition rate, Equation (8), using zero-one loss assignment, for each thematic class $C_{m},\;m=1,2,\ldots ,5,\;$ based on the use of sensor $S_{1}$ Gaussian feature $x_{1}$ only.
- $\tau \left(\pi_{C_{m}}^{G}\right)$: Maximum possibility average pixel-based decision recognition rate, Equation (12), exploiting the epistemic expert knowledge for the description of each considered thematic class $C_{m},\;m=1,2,\ldots ,5,\;$ in the features set $\Theta_{1}$ only.
- $\tau \left(Pr_{C_{m}}^{G}\cdot \pi_{C_{m}}^{R}\right)$: Possibilistic maximum likelihood average pixel-based decision recognition rate, Equation (24), jointly exploiting the epistemic expert knowledge for the description of each considered thematic class $C_{m},\;m=1,2,\ldots ,5,$ in the features set $\Theta_{2}$ (sensor $S_{2}$), and the Gaussian probabilistic knowledge for the description of the same thematic class in the features set $\Theta_{1}$ (sensor $S_{1}$).
- $\tau \left(Pr_{C_{m}}^{R}\right)$: Minimum-risk Bayes average pixel-based decision recognition rate, Equation (8), using zero-one loss assignment, for each thematic class $C_{m},\;m=1,2,\ldots ,5,\;$ based on the use of sensor $S_{2}$ Rayleigh feature $x_{2}$ only.
- $\tau \left(\pi_{C_{m}}^{G}\right)$: Maximum possibility average pixel-based decision recognition rate, Equation (12), exploiting the epistemic expert knowledge for the description of each considered thematic class $C_{m},\;m=1,2,\ldots ,5,\;$ in the features set $\Theta_{1}$ (sensor $S_{1}$).
- $\tau \left(Pr_{C_{m}}^{R}\cdot \pi_{C_{m}}^{G}\right)$: Possibilistic maximum likelihood average pixel-based decision recognition rate, Equation (24), jointly exploiting the epistemic expert knowledge for the description of each thematic class $C_{m},\;m=1,2,\ldots ,5,\;$ in sensor $S_{1}$ features set $\Theta_{1}$, and the Rayleigh probabilistic knowledge for the description of $C_{m},\;m=1,2,\ldots ,5,$ in sensor $S_{2}$ features set $\Theta_{2}$.
- $\tau \left(Pr_{C_{m}}^{G}\cdot Pr_{C_{m}}^{R}\right)$: Minimum-risk Bayes average pixel-based decision recognition rate, Equation (8), using zero-one loss assignment, for each thematic class $C_{m},\;m=1,2,\ldots ,5,\;$ and based on the joint use of both sensors $S_{1}$ (associated with Gaussian probabilistic knowledge in the features set $\Theta_{1}$) and *S*_2_ (associated with Rayleigh probabilistic knowledge in the features set $\Theta_{2}$. Sensors $S_{1}$ and $S_{2}$ are considered as being statistically independent. This criterion as well as all the criteria above have been calculated for the example in Table 2.

The obtained average recognition rates are summarized in Table 2 (last row). As expected, at the global scene level, the average recognition rates when a probabilistic information source is used (for modelling the observed features) are higher than those obtained by the use of epistemic knowledge (i.e., $\tau \left(Pr^{G}\right)\geq \tau \left(\pi^{G}\right)$, and $\tau \left(Pr^{R}\right)\geq \tau \left(\pi^{R}\right)$). Nevertheless, at the thematic classes’ level, this property does not hold for some classes. This is mainly due to the fact that for “sharp classes” probability density functions, i.e., small variance, (for instance, thematic classes $C_{2}$ and $C_{4}$), the possibility distributions shape used to encode the expert knowledge (i.e., a wide-based triangular possibility shape) may bias each class influence, leading to a better recognition rate to the detriment of other neighboring classes (for instance, class $C_{3}$). In this case, this leads to obtain $\;\tau \left(\pi_{C_{2}}^{G}\right)>\tau \left(Pr_{C_{2}}^{G}\right)$ and $\tau \left(\pi_{C_{4}}^{G}\right)>\tau \left(Pr_{C_{4}}^{G}\right)$.

Poorer recognition performances of the maximum possibility decision criterion clearly come from the “weak epistemic knowledge” produced by the expert (indicating just the mean values) compared to the “strong probabilistic” knowledge involved by full probability density functions (resulting from either *a priori* information or the densities estimation using some learning data). The most interesting and promising result can be witnessed in terms of recognition rate improvement when the epistemic knowledge is jointly used with the probabilistic one as proposed by the PML decision criterion. Indeed, Table 2 (columns 4 and 7, bold numbers) shows that for all classes $C_{m},\;m=1,2,\ldots ,5,$ we have $\tau \left(Pr_{C_{m}}^{G}\cdot \pi_{C_{m}}^{R}\right)\geq \tau \left(Pr_{C_{m}}^{G}\right)$ and $\;\tau \left(Pr_{C_{m}}^{R}\cdot \pi_{C_{m}}^{G}\right)\geq \tau \left(Pr_{C_{m}}^{R}\right)$.

It is worthwhile to notice (columns 4 and 7, bold numbers) that the level of performance improvement depends on the “informative” capacity of the “additional” knowledge source. For instance, embedding the Gaussian source of knowledge (in terms of epistemic knowledge form) into the decisional process based on the probabilistic Rayleigh source of knowledge, improves much more the performance level than the reverse (i.e., embedding epistemic Rayleigh source of knowledge into the decisional process based on the probabilistic Gaussian source of knowledge):$\;\tau \left(Pr_{C_{m}}^{G}\cdot \pi_{C_{m}}^{R}\right)\approx$$\tau \left(Pr_{C_{m}}^{G}\right)$ whereas $\;\tau \left(Pr_{C_{m}}^{R}\cdot \pi_{C_{m}}^{G}\right)>\tau \left(Pr_{C_{m}}^{R}\right)$.

Finally, it is important to notice that the PML decision performances are lower-upper bounds delimited as follows:

(30) $$\begin{array}{c} \tau \left(Pr_{C_{m}}^{G}\right)\leq \tau \left(Pr_{C_{m}}^{G}\cdot \pi_{C_{m}}^{R}\right)\leq \tau \left(Pr_{C_{m}}^{G}\cdot Pr_{C_{m}}^{R}\right)\\ \tau \left(Pr_{C_{m}}^{R}\right)\leq \tau \left(Pr_{C_{m}}^{R}\cdot \pi_{C_{m}}^{G}\right)\leq \tau \left(Pr_{C_{m}}^{R}\cdot Pr_{C_{m}}^{G}\right) \end{array}$$

Given the fact that the two sources S_1_ and S_2_ are assumed to be statistically independent, then the joint probability distribution of the augmented feature vector $x=\left[x_{1}\;x_{2}\right]$ is the direct product of marginal ones. This simply means that the upper bounds given in Equation (30) constitute the optimal recognition rate (obtained by considering both probabilistic sources of knowledge). Therefore, the PML criterion improves the performances of the use of a “single” probabilistic source of knowledge, and approaches for some thematic classes the optimal recognition rate upper bound (last column of Table 2).

## 7. Conclusions

In this paper, a new criterion for decision-making process in classification systems is proposed. After a brief recall of the Bayesian decision-making criteria, three major possibilistic decision making criteria, i.e., maximum possibility, maximum necessity measure and confidence index maximization, have been detailed. It was clearly shown that the three considered decision criteria lead to, at best, the maximum possibility decision criterion. However, the maximum possibility criterion has no physical justification. A new criterion called, possibilistic maximum likelihood (PML) framed within the possibility theory, but using notions from Bayesian decision-making, has been presented and its behavior evaluated. The main motivation being the development of such criterion is for multisource information fusion where a pattern may be observed through several channels and where the available knowledge, concerning the observed features, may be of a probabilistic nature for some features, and of an epistemic nature for some others.

In this configuration, most encountered studies transform one of the two knowledge types into the other form, and then apply either the classical Bayesian or possibilistic decision-making criteria. In this paper, we have proposed a new approach called the Possibilistic maximum likelihood (PML) decision-making approach, where the Bayesian decision-making framework is adopted and where the epistemic knowledge is integrated into the decision-making process by defining possibilistic loss values instead of the usually used zero-one loss values.

A set of possibilistic loss values is proposed and evaluated in the context of pixel-based image classification where a synthetic scene, composed of several thematic classes, was randomly generated using two types of sensors: a Gaussian and a Rayleigh sensor. The evaluation of the proposed PML criterion has clearly shown the interest of the application of PML; where the obtained recognition rates approach the optimal rates (i.e., where all the available knowledge is expressed in terms of probabilistic knowledge). Moreover, the proposed PML decision criterion offers a physical interpretation of the maximum possibility decision criterion as a special case of the possibilistic Bayesian decision-making process when all the available probabilistic information indicates equal decisions probabilities.

## Figures and Tables

**Table 1:** PML decision making behavior for several cases.

	**Case 1**			**Case 2**			**Case 3**		
	$Pr\left\{\cdot |x_{1}\right\}$	$\pi_{x_{2}}\left(\cdot \right)$	υ(·)	$Pr\left\{\cdot |x_{1}\right\}$	$\pi_{x_{2}}\left(\cdot \right)$	υ(·)	$Pr\left\{\cdot |x_{1}\right\}$	$\pi_{x_{2}}\left(\cdot \right)$	υ(·)
*C*_1_	0.1	0.2	0.02	0.1	1.0	0.1	0.2	0.8	0.16
*C*_2_	0.4	0.7	0.28	0.4	1.0	0.4	0.2	0.2	0.04
*C*_3_	0.1	0.3	0.03	0.1	1.0	0.1	0.2	0.1	0.02
*C*_4_	0.3	0.7	0.21	0.3	1.0	0.3	0.2	0.1	0.02
*C*_5_	0.1	0.1	0.10	0.1	1.0	0.1	0.2	0.2	0.04
	**Case 4**			**Case 5**			**Case 6**		
	$Pr\left\{\cdot |x_{1}\right\}$	$\pi_{x_{2}}\left(\cdot \right)$	υ(·)	$Pr\left\{\cdot |x_{1}\right\}$	$\pi_{x_{2}}\left(\cdot \right)$	υ(·)	$Pr\left\{\cdot |x_{1}\right\}$	$\pi_{x_{2}}\left(\cdot \right)$	υ(·)
*C*_1_	0.1	1.0	0.1	0.1	0.8	0.08	0.1	0.8	0.08
*C*_2_	0.4	0.2	0.08	0.4	0.2	0.08	0.4	0.3	0.12
*C*_3_	0.1	0.0	0.0	0.1	0.1	0.01	0.1	0.4	0.04
*C*_4_	0.3	0.0	0.0	0.3	0.3	0.03	0.35	0.5	0.16
*C*_5_	0.1	0.0	0.0	0.1	0.1	0.02	0.1	0.5	0.05

**Table 2:** PML decision average pixel-based recognition rates for the five thematic classes using various configurations of knowledge sources.

Knowledge Sources	Source *S*_1_: Probabilistic (G: Gaussian) Source *S*_2_: Epistemic (Expert)			Source *S*_2_: Probabilistic (R: Rayleigh) Source *S*_1_: Epistemic (Expert)			Both Sources Are Probabilistic
	$S_{1}$	$S_{2}$	$S_{1}\underset{\mathrm{PML}}{\underset{︸}{⊕}}S_{2}$	$S_{2}$	$S_{1}$	$S_{1}\underset{\mathrm{PML}}{\underset{︸}{⊕}}S_{2}$	$S_{1}⊕S_{2}$
Criterion	$\tau \left(Pr_{C_{m}}^{G}\right)$	$\tau \left(\pi_{C_{m}}^{G}\right)$	$\tau \left(Pr_{C_{m}}^{G}\cdot \pi_{C_{m}}^{R}\right)$	$\tau \left(Pr_{C_{m}}^{R}\right)$	$\tau \left(\pi_{C_{m}}^{G}\right)$	$\tau \left(Pr_{C_{m}}^{R}\cdot \pi_{C_{m}}^{G}\right)$	$\tau \left(Pr_{C_{m}}^{G}\cdot Pr_{C_{m}}^{R}\right)$
*C_1_*	0.96	0.33	0.96	0.34	0.96	0.64	0.96
*C_2_*	0.90	0.32	0.91	0.49	0.98	0.68	0.92
*C_3_*	0.78	0.91	0.81	0.89	0.67	0.90	0.91
*C_4_*	0.94	0.25	0.95	0.32	0.96	0.61	0.97
*C_5_*	0.83	0.72	0.86	0.58	0.75	0.71	0.94
**Average** **Recognition** **Rate**	0.88	0.51	0.90	0.52	0.86	0.71	0.94

## Data Availability

Not applicable.

## References

[1] Dubois D. (2008). Uncertainty theories: A unified view. SIPTA School 08—UEE 08.

[2] Klir G.J., Wierman M.J. (1999). Uncertainty-Based Information: Elements of Generalized Information Theory.

[3] Denoeux T. (2016). 40 years of Dempster-Shafer theory. Int. J. Approx. Reason..

[4] Yager R.R., Liu L. (2008). Classic Works of the Dempster-Shafer Theory of Belief Functions.

[5] Shafer G. (1976). A Mathematical Theory of Evidence.

[6] Zadeh L.A. (1965). Fuzzy sets. Inf. Control.

[7] Zadeh L. (1978). Fuzzy Sets as the Basis for a Theory of Possibility. Fuzzy Sets Syst..

[8] Zadeh L.A. (2006). Generalized theory of uncertainty (GTU)—principal concepts and ideas. Comput. Stat. Data Anal..

[9] Dubois D., Prade H. (2015). The legacy of 50 years of fuzzy sets: A discussion. Fuzzy Sets Syst..

[10] Duda R.O., Hart P.E., Stork D.G. (2012). Pattern Classification.

[11] Solaiman B., Bossé É. (2019). Possibility Theory for the Design of Information Fusion Systems.

[12] Frélicot C. (1998). On unifying probabilistic/fuzzy and possibilistic rejection-based classifiers. Advances in Pattern Recognition.

[13] Solaiman B., Bossé E., Pigeon L., Guériot D., Florea M.C. (2015). A conceptual definition of a holonic processing framework to support the design of information fusion systems. Inf. Fusion.

[14] Tou J.T., Gonzalez R.C. (1974). Pattern Recognition Principles.

[15] Dubois D., Prade H. (1988). Possibility Theory: An Approach to Computerized Processing of Uncertainty.

[16] Dubois D.J. (1980). Fuzzy Sets and Systems: Theory and Applications.

[17] Dubois D., Prade H. (1992). When upper probabilities are possibility measures. Fuzzy Sets Syst..

[18] Aliev R., Pedrycz W., Fazlollahi B., Huseynov O.H., Alizadeh A., Guirimov B. (2012). Fuzzy logic-based generalized decision theory with imperfect information. Inf. Sci..

[19] Buntao N., Kreinovich V. (2011). How to Combine Probabilistic and Possibilistic (Expert) Knowledge: Uniqueness of Reconstruction in Yager’s (Product) Approach. Int. J. Innov. Manag. Inf. Prod. (IJIMIP).

[20] Coletti G., Petturiti D., Vantaggi B. (2014). Possibilistic and probabilistic likelihood functions and their extensions: Common features and specific characteristics. Fuzzy Sets Syst..

[21] Fargier H., Amor N.B., Guezguez W. (2012). On the complexity of decision making in possibilistic decision trees. arXiv.

[22] Guo P. (2007). Possibilistic Decision-Making Approaches. The 2007 International Conference on Intelligent Systems and Knowledge Engineering.

[23] Weng P. (2012). Qualitative decision making under possibilistic uncertainty: Toward more discriminating criteria. arXiv.

[24] Yager R.R. (2011). A measure based approach to the fusion of possibilistic and probabilistic uncertainty. Fuzzy Optim. Decis. Mak..

[25] Yager R.R. (2011). On the fusion of possibilistic and probabilistic information in biometric decision-making. Proceedings of the 2011 IEEE Workshop on Computational Intelligence in Biometrics and Identity Management (CIBIM).

[26] Bouyssou D., Dubois D., Prade H., Pirlot M. (2013). Decision Making Process: Concepts and Methods.

[27] Dubois D., Foulloy L., Mauris G., Prade H. (2004). Probability-possibility transformations, triangular fuzzy sets, and probabilistic inequalities. Reliab. Comput..

[28] Dubois D., Prade H. (1998). Possibility theory: Qualitative and quantitative aspects. Quantified Representation of Uncertainty and Imprecision.

[29] Dubois D., Prade H. (1997). The three semantics of fuzzy sets. Fuzzy Sets Syst..

[30] Dubois D., Prade H., Sandri S. (1993). On possibility/probability transformations. Fuzzy Logic.

[31] Denœux T. (2000). Modeling vague beliefs using fuzzy-valued belief structures. Fuzzy Sets Syst..

[32] Denœux T. (2011). Maximum likelihood estimation from fuzzy data using the EM algorithm. Fuzzy Sets Syst..

[33] Denoeux T. (2013). Maximum likelihood estimation from uncertain data in the belief function framework. Knowl. Data Eng..

[34] Walley P. (1991). Statistical Reasoning With Imprecise Probabilities.

[35] Walley P. (2000). Towards a unified theory of imprecise probability. Int. J. Approx. Reason..

[36] Linda O., Manic M., Alves-Foss J., Vollmer T. (2011). Towards resilient critical infrastructures: Application of Type-2 Fuzzy Logic in embedded network security cyber sensor. Proceedings of the 2011 4th International Symposium on Resilient Control Systems (ISRCS).

[37] Mendel J.M., John R.I.B. (2002). Type-2 fuzzy sets made simple. Fuzzy Syst..

[38] Ozen T., Garibaldi J.M. Effect of type-2 fuzzy membership function shape on modelling variation in human decision making. Proceedings of the IEEE International Conference on Fuzzy Systems.

[39] Luce R.D., Raiffa H. (1957). Games and Decisions.

[40] Haouari B., Amor N.B., Elouedi Z., Mellouli K. (2009). Naïve possibilistic network classifiers. Fuzzy Sets Syst..

[41] Benferhat S., Tabia K. (2008). An efficient algorithm for naive possibilistic classifiers with uncertain inputs. Scalable Uncertainty Management.

[42] Bounhas M., Hamed M.G., Prade H., Serrurier M., Mellouli K. (2014). Naive possibilistic classifiers for imprecise or uncertain numerical data. Fuzzy Sets Syst..

[43] Bounhas M., Mellouli K., Prade H., Serrurier M. (2013). Possibilistic classifiers for numerical data. Soft Comput..

[44] Kikuchi S., Perincherry V. Handling Uncertainty in Large Scale Systems with Certainty and Integrity. http://citeseerx.ist.psu.edu/viewdoc/summary?doi=10.1.1.132.7069.

[45] Yager R.R. (2011). Set measure directed multi-source information fusion. Fuzzy Syst..

[46] Yager R.R. Hard and soft information fusion using measures. Proceedings of the 2010 IEEE International Conference on Intelligent Systems and Knowledge Engineering.

